# Cognitive Impairment Induced by Lead Exposure during Lifespan: Mechanisms of Lead Neurotoxicity

**DOI:** 10.3390/toxics9020023

**Published:** 2021-01-28

**Authors:** Daniela Ramírez Ortega, Dinora F. González Esquivel, Tonali Blanco Ayala, Benjamín Pineda, Saul Gómez Manzo, Jaime Marcial Quino, Paul Carrillo Mora, Verónica Pérez de la Cruz

**Affiliations:** 1 Laboratorio de Neurobioquímica y Conducta, Instituto Nacional de Neurología y Neurocirugía Manuel Velasco Suárez, S.S.A., México D.F. 14269, Mexico; drmz_ortega@hotmail.com (D.R.O.); dinora.gonzalez@innn.edu.mx (D.F.G.E.); tonaliblaya@gmail.com (T.B.A.); 2 Laboratorio de Neuroinmunología, Instituto Nacional de Neurología y Neurocirugía Manuel Velasco Suárez, S.S.A., México D.F. 14269, Mexico; benjamin.pineda@innn.edu.mx; 3 Laboratorio de Bioquímica Genética, Instituto Nacional de Pediatría, Secretaría de Salud, México City 04530, Mexico; saulmanzo@ciencias.unam.mx; 4 CONACYT—Instituto Nacional de Pediatría, Secretaría de Salud, Ciudad de México 04530, Mexico; jmarcialq@ciencias.unam.mx; 5 División de Neurociencias, Instituto Nacional de Rehabilitación, S.S.A., México D.F. 14389, Mexico; neuropolaco@yahoo.com.mx

**Keywords:** heavy metals, lead, cognition, neurotoxicity

## Abstract

Lead (Pb) is considered a strong environmental toxin with human health repercussions. Due to its widespread use and the number of people potentially exposed to different sources of this heavy metal, Pb intoxication is recognized as a public health problem in many countries. Exposure to Pb can occur through ingestion, inhalation, dermal, and transplacental routes. The magnitude of its effects depends on several toxicity conditions: lead speciation, doses, time, and age of exposure, among others. It has been demonstrated that Pb exposure induces stronger effects during early life. The central nervous system is especially vulnerable to Pb toxicity; Pb exposure is linked to cognitive impairment, executive function alterations, abnormal social behavior, and fine motor control perturbations. This review aims to provide a general view of the cognitive consequences associated with Pb exposure during early life as well as during adulthood. Additionally, it describes the neurotoxic mechanisms associated with cognitive impairment induced by Pb, which include neurochemical, molecular, and morphological changes that jointly could have a synergic effect on the cognitive performance.

## 1. Introduction

Lead (Pb) is a toxic heavy metal found in different sources that include contaminated drinking water, batteries, gasoline, paint, food cans, traditional folk remedies, water pipes, Pb-glazed ceramics, Pb-crystal, cosmetics, jewelry, cigarette smoke, Pb-solder used to produce vinyl lunch boxes and children’s toys, and even contaminated candy [1,2]. As a consequence of its environmental persistence, transportability, and widespread sources, Pb is considered a public health problem of global magnitude. Among the countries with a major prevalence of Pb toxicity in humans are India, Indonesia, the Philippines, Nigeria, China, Pakistan, Brazil, Mexico, Peru, France, and the United States [3,4]. The main route of Pb entry into the organism is oral; the gastrointestinal tract absorbs around 5–15% of Pb and the rest is excreted in the feces. Some Pb particles are inhaled and by ciliary action of respiratory epithelial cells these are swallowed into the gastrointestinal tract. There are two types of Pb in the body: diffusible (mobile) and non-diffusible (fixed) forms [5]. Mobile Pb is considered biologically active and can be transported from one part of the body to another, while fixed Pb is accumulated in soft tissues (intestine, lungs, liver, spleen, kidney, and central nervous system (CNS)) and bones [6]. The excretion of Pb is carried out by the liver through bile secretion and by the kidneys through glomerular filtration and transtubular flow [7]. The Pb half-life in the bloodstream is around 35 days; however, once absorbed, it can be dispersed to soft tissues and stored in the bones for extended periods of time with estimations being around 30 years [8]. Pb is highly permeable and can cross the placental and blood–brain barrier (BBB) and can also be present in breast milk. According to data research concerning Pb poisoning long-term effects, Pb toxic threshold has been drastically reduced from 60 µg/dL in the 1960s to 10 µg/dL in 1991. Currently, the Center for Disease Control and Prevention (CDC) has established ≥5 μg/dL as blood Pb reference value; however, there are no measurable blood levels of Pb found to be safe since even low levels of Pb produce toxic effects (Table 1) [9,10]. 

Clinical effects of Pb poisoning include neurological features such as encephalopathy, headache, seizures, cerebral palsy, and confusion; renal failure and convulsions can also appear. As Pb exposure increases, severe effects of Pb can be lethal. The severity of these effects is directly associated with the concentration of Pb in the blood [7,20]. In this context, several studies describe some factors that confer susceptibility to Pb toxicity: age, metabolism of calcium, phosphorus, iron, vitamins, ascorbic acid, dietary protein, alcohol consumption, pregnancy, and co-existent diseases [7]. 

**Figure 1:**
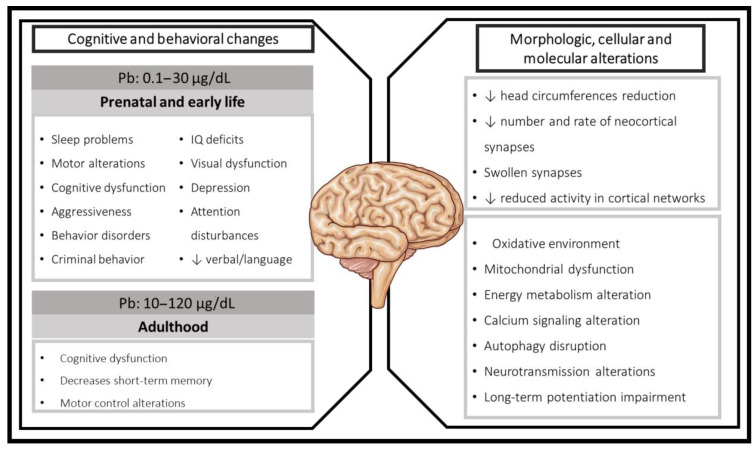
Cognitive and behavioral changes induced by Pb exposure in humans and associated morphologic, cellular, and molecular alterations of Pb toxicity.

According to the World Health Organization (WHO), the fetal stage of human development and young children are particularly vulnerable to Pb toxicity mainly because of a higher absorption rate of 4–5 times as much ingested Pb as adults from a given source [21,22]. The CNS is one of the targets where Pb toxicity exerts its most harmful and long-term effects, being especially severe if the exposure occurs during early life [20]. In this context, Gulson and coworkers showed that Pb mobilization to the fetus or newborn children can occur from endogenous sources, such as the maternal skeleton during pregnancy and in higher concentrations during lactation due to Pb ability to pass through the mammary gland barrier and the increased physiologic demand for calcium during this period of time [23,24,25]. There is evidence suggesting that Pb exposure causes severe effects in cognitive function in both adults and children, including intellectual and learning disabilities and behavioral disorders [1]. These deleterious effects have been associated with several molecular and cellular mechanisms including alterations in DNA and chromosomal integrity [26]; Pb direct interaction with proteins, especially with those presenting metal-binding sites due to its high electronegativity [26]; and alteration to cellular redox status considering that Pb can generate reactive oxygen species (ROS) simultaneously with depletion of antioxidants systems due to its high affinity for thiol groups [27] (Figure 1). Herein, we provide an overview of the factors described in the literature that can converge and influence the cognitive alterations induced by Pb exposure both in humans and experimental models. First, we describe the cognitive alterations found in humans exposed to Pb during early life and in adulthood. Then, we discuss the mechanisms involved in Pb neurotoxicity and the experimental therapies used to counteract, mainly the redox disbalance and cognitive impairment induced by Pb.

## 2. Lead Exposure during Developing Brain, Early Life, and Adulthood: Clinical Evidence

The human brain, in prenatal and early postnatal periods, undergoes rapid growth and is extremely sensitive to environmental pollutants including heavy metals such as Pb [28]. As mentioned above, Pb produces severe effects in the CNS and particularly in the developing brain, due to the immature blood–brain barrier and the high rates of cellular proliferation, differentiation, and synaptogenesis distinctive of this period. 

### 2.1. Cognitive Effects Associated with Prenatal and Early Postnatal Pb Exposure in Humans

Lead poisoning has been reported in the entire population, from newborns to adults (Table 2). In the adult body, over 90% of Pb is stored in the bones and teeth; however, it can reenter the blood and soft tissues during periods of heightened bone turnover [29,30,31]. Nonetheless, the developing brain is more sensitive to Pb because the fetal blood–barrier is more permeable, and the fetus bone’s capacity for sequestering this heavy metal is reduced compared to adults. In the fetus, the placenta is the first source of Pb exposure due to Pb distribution into maternal blood and later easy transportation through the placenta. It has been estimated from umbilical cord blood samples that the fetal blood Pb levels correlate (80–90%) with those found in maternal blood [32,33,34]. Pb can also be found in the breast milk, thus representing an additional source of exposure for newborns [32,35,36,37,38,39]. A correlation between Pb concentration in blood and maternal milk of 53 pregnant women and newborns blood samples was established, suggesting that Pb transport is nonselective; hence, the placenta and blood-brain barrier allow Pb passage through passive and facilitated diffusion [40,41].

Numerous factors can contribute to the severity of Pb toxic effects: nutritional status, smoking, occupational environment, etc. It has been demonstrated that calcium deficiency increased Pb absorption [61,62]. In this context, high blood Pb levels have been related to moderate (hemoglobin: 7.0–9.9 g/dL) and severe (hemoglobin < 7.0 g/dL) anemia in pregnant women. Furthermore, even low blood Pb levels are causally related to an elevated risk of mild and moderate Fe deficiency anemia [63]. An oxidant environment induced by Pb exposure in anemic pregnant women also exhibited a strong correlation between blood Pb concentration and redox biomarkers such as catalase (CAT) and superoxide dismutase (SOD) enzymatic activities along with lipid peroxidation products were characterized [63]. Interestingly, an additional study found a weak correlation between the concentration of Pb in the neonatal blood and the concentration of vaccenic acid (a fatty acid and oxidative stress inducer) in maternal blood [41,64]. 

Lead exposure during neurodevelopment is especially damaging because this metal can trigger some irreversible alterations in the structure and function of the CNS. A recent study based on the Barley cognitive scores obtained in 24-month-old children showed that the psychosocial stress observed during the pregnancy has an additive harmful effect together with the blood Pb levels on neurodevelopment [65]. Additionally, as revealed by a longitudinal study analyses in newborns where a direct correlation between elevated cord blood Pb levels (above 2.475 µg/dL) and head circumferences reduction was found, this could represent a critical factor in long-term cognitive deficits [66]. Alterations in the pattern of arousal states in newborns are also affected by Pb exposure and have been suggested to be useful for risk stratification; Pb levels higher than 10 µg/dL in umbilical cord blood correlated with arousal states characterized by restless, fidgety, increased motor activity, and intense crying in newborns, indicating that they were least likely to transition from awake states back into deep or light sleep states [67]. Another study conducted in Mexico City in 24-month-old infants found that Pb levels in both umbilical cord blood and maternal trabecular bone were inversely associated with the Mental Development Index (MDI) scores of the Bayley Scale [68]. Similarly, an additional study in two-year-old children previously characterized with high Pb cord blood levels at birth (10–25 µg/dL) had lower scores in the cognitive development test (MDI); such effect was pronounced from birth to two years of age, but undetectable at 57 months. Based on this last finding, Bellinger et al. proposed that this later recovery was due to sociodemographic factors such as additional cognitive or psychosocial stimulation [69,70]. 

Moreover, a Yugoslavian prospective study in three-, four-, five- and seven-year-old children from mothers recruited during pregnancy from a smelter town confirmed that prenatal and postnatal early life Pb exposure is associated with a lower intelligence quotient (IQ) score [71]. Within the same country, a similar cohort study in children (4.5 years old) showed a modest association between early Pb exposure and fine motor and visual-motor functioning [72]. Lead exposure has also been associated with attention-deficit/hyperactivity disorder (ADHD) in children [73,74,75,76,77]. Recently, it was shown that prenatal Pb exposure causes prepulse inhibition (PPI) deficits in children and adolescents, indicating an inadequate organization in the cognitive process [78]. In addition, it has been shown that a higher cumulative Pb level was associated with higher sleep fragmentation in adolescents [79].

### 2.2. Cognitive Impairments in Children Pb-Exposed

Children form part of the most vulnerable sector to Pb poisoning due to the frequency of hand-to-mouth activity and a higher calcium demand for their growth. Lead mimics calcium; thus, this heavy metal, even at low concentrations, has severe effects in the growing children. Children are more vulnerable to Pb exposure than adults because they absorb 40–50% of dietary Pb, whereas adults absorb only 5–10% [80]. An additional concern about exposure to Pb in this age group are the candies wrapped with decorative paints with Pb content that are commonly consumed at this age [81]. In countries such as Mexico and USA, consumption of these candies has been related to an increase in blood Pb levels in children 2–6 years of age. Although this source of exposure appears to be mild, this should be studied in greater detail in the future because it may represent a source of mild but chronic Pb exposure in children [82,83]. Another important source of Pb exposure in children includes folk remedies [81]. In this context, there are reports of children suffering with acute Pb poisoning after they were treated for stomachache or intestinal illness with traditional medicines such as greta and/or azarcon (folk remedies containing Pb oxide and Pb tetroxide, respectively) [84,85]. 

The effect of Pb on cognitive parameters has been widely reported (Table 1), especially in memory and learning [10,86,87]. Cognitive long-term deficits were evaluated in children from a Pb smelter community; the evaluation started at two years old with blood concentrations of 30 µg Pb/dL and a deficit of 3.3 points on the Bayley Mental Development Index was found; at the age of four, they had a deficit of 7.2 points on the McCarthy General Cognitive Index that persisted until the age of seven years old. This evidence suggested that Pb exposure in the preschool age has a maximal detrimental effect on IQ [86,87]. Another study in children, in whom Pb levels were determined in the teeth as a principal indicator for long-term cumulative Pb exposure, was performed. Pb was measured and the children were separated into two groups: low 4.6 µg/g (range: 1.4–12.7 µg/g) and high 6.2 µg/g (range: 1.9–38.5 µg/g) Pb levels. The group with the highest Pb levels in the teeth showed deterioration of visual-motor integration and IQ deficits (5–7 points) along with deficits in verbal IQ (4–7 points) and full-scale IQ. Moreover, the parents or teachers of the children with the highest Pb concentrations indicated that the children were easily distracted, restless, and they lacked the interest to do their homework [13]. Protein, zinc, iron, and calcium deficiency are additional consequences of Pb exposure in children. Evidence from high and low Pb-polluted areas showed that children living in high-risk areas of Pb pollution had Pb levels ≥ 10 µg/dL; 37% of these children showed cognitive dysfunction, which was also associated with lower hemoglobin (anemia) unlike those without cognitive impairment [14]. In agreement, a study focused on Mexican children aged 6–8 years (*n* = 602) living close to a metal factory found that 50% of the study population had Pb blood levels ≥10 µg/dL. The prevalence of anemia (hemoglobin <12.4 g/dL) and zinc and iron deficiency were 10%, 28.9%, and 21.3%, respectively. Children with higher Pb levels were associated with earlier bedtime and fewer sleep hours than children with lower Pb levels. Additionally, it was demonstrated that each 1 µg/dL increase in Pb blood levels was positively associated with lower physical activity [88]. Consistent with these reports, a recent study in 2–4-year-old children (named as the most vulnerable age for Pb toxicity since it appears to be a critical period for intelligence and academic achievement), showed that blood Pb levels were negatively correlated with development quotients of adaptative behavior, gross and fine motor performance, language development, and individual social behavior [11]. Motor dysfunction is an additional detrimental effect of Pb exposure. Bhattacharya et al. showed that low to moderate Pb exposure (~5.9 μg/dL) in early childhood induces impairment on the maturation of postural balance, while higher Pb levels (6.5 μg/dL) have been associated with more severe motor dysfunction including problems with postural balance, gait, and locomotor activities [89,90].

A study in 1979 determined that children with high dentine Pb levels showed a lower score on the Wechsler Intelligence Scale for Children compared with children with low dentine Pb levels. In particular, behavioral effects such as the frequency of non-adaptive classroom behavior was related to dentine Pb levels [91]. In this context, a retrospective cohort study showed that high-Pb bone subjects (7–11 years old) were more likely to obtain worse scores regarding a self-reported antisocial behavior scales with a strong association between higher Pb bone levels and an increased risk for aggression and delinquency behaviors in 11-year-old boys [92]. Furthermore, it was demonstrated that cumulative prenatal Pb exposure increased the likelihood to exhibit difficult temperament, this effect being even higher when the mother had higher prenatal depression scores [93]. Notably, maternal self-esteem is associated with better neurodevelopmental test scores in Pb-exposed children [94]. Supporting this evidence, a study based on structural equation models showed that the presence of an enriched home environment, for instance maternal support for the child’s schoolwork and extracurricular activities, seems to moderate the effects of Pb in cognition and behavior on first-grade elementary school children living in a Mexican Pb smelter community [95].

Biochemical changes have also been characterized in those children exposed to a source of Pb intoxication. In the case of children who have some contact with a source of Pb exposure in mechanic or painting workshops high Pb levels in the blood (>5 µg/dL) were found; additionally, they presented a blood increase of malondialdehyde (MDA) levels and a decrease in vitamin E levels compared with the children who had Pb blood levels less than 5 µg/dL, suggesting the prevalence of oxidative environment induced by Pb exposure [96]. Similarly, a study carried out in children (3–12 years) with neurological disorders (cerebral palsy, seizures, and encephalopathy) showed that blood Pb levels were significantly higher in children with seizures (15.52 µg/dL), cerebral palsy (17.97 g/dL), and encephalopathy (24.51 µg/dL) compared with the control group (10.37 µg/dL). Additionally, higher Pb levels in the blood had an increase in MDA and a reduction in GSH levels and δ-aminolevulinic acid dehydratase activity [20]. Among the consequences of Pb exposure, alterations in cellular metabolism have also been reported. Meng and coworkers showed, using ^1^H magnetic resonance spectroscopy (MRS), a reduction in neuronal density, mitochondrial and phosphate metabolism, and a membrane turnover in four brain regions (right and left frontal, left and right hippocampus) from Pb-exposure children when compared to same age matched-controls [97].

### 2.3. Adulthood Pb Exposure

Although the effect of Pb is more severe in children, the consequences can be persistent even in adulthood. In this context, 35 adults exposed to Pb during the first four years of childhood were evaluated 50 years after poisoning. The subjects exposed to Pb during childhood had an inferior performance in cognitive tasks when they were compared against the control group; notably, their occupational status was related to their deficit in the neuropsychological functioning of everyday life. Therefore, this study suggested that childhood exposure to Pb can be sufficient to produce cognitive deficits in adulthood, despite the cessation of Pb exposure [8]. 

As mentioned above, Pb exposure during early life has severe effects on cognitive functions, but it also has effects during adulthood, mainly due to occupational activity and environmental exposure. It is important to consider that elderly people nowadays were exposed to high levels of Pb in the environment due to the extensive use of this metal in the past. In this context, it has been shown that a higher osseous Pb content is associated with worse cognitive function in community-dwelling elderly adults (50–70 years). This association was shown to be diminished after an adjustment regarding years of education, wealth, and race/ethnicity [98]. Additionally, osseous Pb concentration was associated with a higher risk for hypertension in older people [99]. Interestingly, when a similar population was evaluated for the additional impact of the neighborhood psychosocial hazards on the Pb-cognitive effects, it was found that the association between osseous Pb and cognitive dysfunctions is exacerbated by the environmental stress [1]. Additionally, it has been found that olfactory recognition deficits are associated with cumulative Pb exposure in a cohort of elderly men from a Boston area community [100]. Similar effects were found in industrial chemical workers (mean age: 41.3 ± 7.8 years; length of Pb exposure: 8.38 ± 6 years), who showed a worse olfactory function compared with the controls [101].

A descriptive study performed in retired former female workers who had worked in plants producing Pb batteries showed reduced activity in distributed cortical networks, compared to the control group. In addition, Pb-exposed workers showed a reduced activation in the dorsolateral prefrontal cortex and the ventrolateral prefrontal cortex compared to controls, suggesting that memory deficits could be attributable to the deficient neural activation because of Pb exposure [19]. According to this evidence, it was shown by MRS using the metabolic marker ratio N-acetylaspartate:creatinine that chronic Pb-exposure cause neuronal loss and correlates with the working memory/executive dysfunction in retired painters twin brothers (71 years old; bone Pb: Twin 1: 343 + 9.4 µg/g and Twin 2: 119 + 8.8 µg/g) [18]. An additional study in workers from a battery recycling plant showed that occupational Pb exposure results in impairment of certain cognitive abilities such as executive functions and short-time memory in workers with moderate to high Pb levels (24–76 µg of Pb/dL) [102].

In addition to the cognitive alterations described, there are reports where Pb levels, present in the blood or bones, have been related to the development of pathologies such as depression, bipolar disorder, anxiety disorders, or schizophrenia in adults [103,104,105,106]. However, most of these associations are only epidemiological, and more studies are necessary to understand the role of Pb in the pathophysiology of these disorders [107]. On the other hand, although there is evidence that chronic Pb poisoning seems to be related to decreased cognitive performance, there are still no formal longitudinal epidemiological studies that demonstrate an association between previous exposure to Pb and the future risk of developing Alzheimer’s disease or other dementias, but experimental studies seem to show that multiple mechanisms of damage are common between Pb-neurotoxicity and Alzheimer’s disease [108].

As mentioned above, environmental and occupational exposure to inorganic Pb persists to be a serious public health problem mainly because: (1) even at lower concentrations Pb produces several cognitive consequences; and (2) elderly people represent the population mostly chronically exposed to Pb, and thus they may be more prone to present abnormal cognitive aging performance due to the accumulation of Pb. The challenge is to identify the mechanisms by which this metal exerts its toxic actions in the CNS and then integrate them, in order to develop potential strategies to counteract these harmful effects and improve long-term cognitive development. To accomplish this latter purpose, these mechanisms have been extensively studied through diverse experimental models, both in vivo and in vitro, and they are summarized in the following sections. 

## 3. Mechanisms Related to Cognitive Impairment Induced by Pb Exposure in Experimental Models

**Figure 2:**
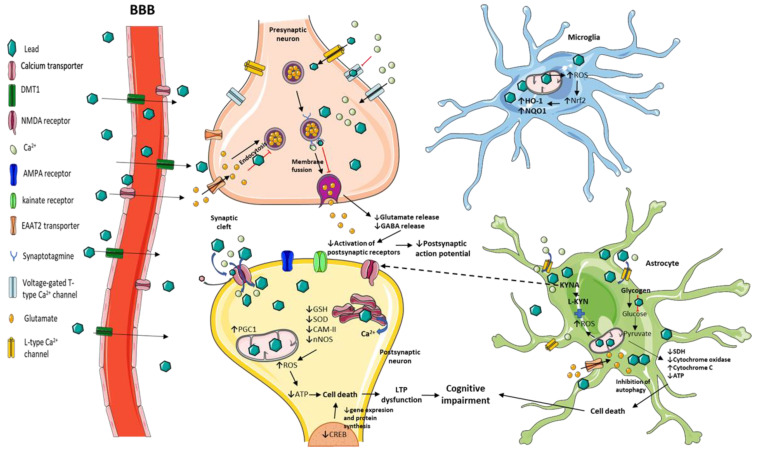
Mechanisms involved in Pb toxicity in the CNS. Pb can enter the CNS through DMT1 and calcium transporters. In the presynaptic neuron, Pb binds with greater affinity to voltage-gated calcium channels and decreases transportation of calcium ions. Through these channels, Pb can cross inside the cell. Once inside, Pb interacts through Ca^2+^ binding sites, with several neuronal components involved in vesicular mobilization and docking, affecting the vesicular mobilization and the neurotransmitter release, thus decreasing the activation of postsynaptic receptors. Pb can form Pb–NMDA complexes altering the intracellular levels of Ca^2+^ in the postsynaptic neuron. The kynurenic acid produced in the astrocytes and rise by Pb contributes to LTP dysfunction. Finally, Pb alters the redox environment, promoting an oxidant environment and cell death.

Although it is well established that even low Pb exposure levels result in long-lasting detrimental effects on children’s intellectual and cognitive function, the mechanisms involved in this process are not completely clear. However, several experimental models provide evidence of cellular mechanisms and molecular targets, generating therapeutic opportunities. Experimental studies conceived to know more about the effects and mechanism involved in Pb toxicity, specifically in the CNS, have been implemented aiming to establish causality between the cognitive dysfunction induced by this heavy metal with converging factors such as redox and energetic homeostasis alterations, calcium dynamics and signaling variations, neurotransmission changes, and morphological and histological alterations (Figure 2). All of these are suggested as potential new targets to ameliorate Pb neurotoxicity.

### 3.1. Neurotransmission and Long-Term Potentiation (LTP) Impairment by Pb Toxicity

The precise mechanisms that explain deleterious effects of Pb on cognition during the neurodevelopment are diverse and not fully addressed yet. However, experimental studies targeting the NMDA receptor (NMDAr) and thus its central role in brain development, synaptic plasticity, and learning and memory have produced wide-ranging literature [109]. Extensive in vitro evidence from neural cell cultures have shown that Pb is a selective and potent non-competitive antagonist of the NMDAr [110,111,112,113]. Additionally, a differential regulation in NMDAr subunits has been reported in animals exposed to Pb during early stages of life; Pb increases hippocampal NR1 subunit expression (at 14 and 21 days old) and downregulates NR2A subunit expression in the pyramidal and granule cell layers of the hippocampus; these disruptions may induce long-lasting changes on hippocampal synaptic plasticity triggering performance impairments seen in spatial learning task of rats exposed to Pb during early life [114]. As mention above, NMDAr subunits play critical roles in hippocampal synaptic plasticity and their alterations are associated with a defective long-term potentiation (LTP) process [115]. Toscano and co-workers showed that Pb exposure during rat development results in an increased expression of NR2B-containing NMDAr complex, thus maintaining a greater proportion of NMDAr complexes in an NR1/NR2B immature form similar to those found in nascent synapses. Therefore, Pb prevents the switch of NR2B- to NR2A-containing NMDAr complexes and results in altered excitatory postsynaptic current amplitude, a decrease in the calcium influx, and impaired synaptic plasticity [116].

In addition to this experimental evidence, Pb can alter the LTP through interference with neurotransmitter systems, particularly those that depend on cellular calcium, since Pb acts as a calcium mimic therefore altering all calcium-dependent processes [117,118]. It has been demonstrated that Pb decreases GABA release in developing rat hippocampal slices possibly by presynaptic calcium channels blocked [119]. Neurotransmission of glutamate is also altered by Pb through its selective-blockade action on presynaptic N-type Ca^2+^ channels, hence reducing the action potential-dependent release of both glutamate and GABA from hippocampal neurons [120]. Furthermore, Pb exposure reduces both voltage-activated calcium channel currents (VACCCs) and NMDA-activated channel currents (NACCs) through the receptor/channel complex in cultured rat dorsal root ganglion neurons [121]. Conversely to the extracellular effect of Pb on calcium channels and considering that Pb transport is through the L-type calcium channels and can be accumulated within the cells, it has been demonstrated in bovine adrenal chromaffin cells that Pb can also alter calcium channel activity intracellularly [122]. Intracellular Pb promotes Ca^2+^ currents by attenuating the Ca^2+^-dependent steady state inactivation of calcium channels, therefore disrupting calcium signaling after chronic exposure of Pb intracellularly [123]. Experimental evidence from in vitro experiments using hippocampal synaptosomes demonstrated that the spontaneous neurotransmitter release of acetylcholine (Ach) induced by Pb is apparently due to either an increase in intraneuronal ionized calcium or the stimulation of Ca^2+^-activated molecules mediating neurotransmitter release [124]. According to this idea, it has been described that Pb affects cellular mechanism related to neurotransmitter release. This hypothesis was supported by experimental evidence using Pb at nanomolar concentration showing that Pb can bind to the same site as Ca^2+^ in the synaptic vesicle-associated to protein synaptotagmin 1 consequently inhibiting membrane fusion and interfering with neurotransmitter release [125]. Additionally, in vitro experiments using isolated synaptic vesicles from rat brains showed that Pb (10 µM) enhanced calmodulin-dependent synaptic vesicle protein phosphorylation promoting acetylcholine release. However, ex-vivo experiments starting from Pb-exposed animals showed a reduction of acetylcholine release mediated by Ca^2+^/calmodulin in synaptic vesicles isolated from these animals [126]. The calcium/calmodulin-dependent protein kinase II (CaM kinase II), a kinase that phosphorylates associated-synaptic proteins, is also a Pb target. Toscano and co-workers demonstrated that rats exposed to Pb during development decreased in 41% CaM kinase II activity and suggested an association with learning and memory impairments seen in this in vivo model [127]. Furthermore, evidence supporting alterations in presynaptic neurotransmitter release as a consequence of Pb exposure has also been demonstrated using hippocampal neurons. Pb exposure during synaptogenesis resulted in an increase of the number of nascent presynaptic docking sites; however, many of these presynaptic contact sites lack the soluble NSF-attachment protein receptor complex involved in vesicular exocytosis. Pb also reduces the expression of vesicular proteins critical for vesicular contact and release (synaptophysin and synaptobrevin), leading to an impaired vesicular release and a reduction of the number of fast-releasing sites in glutamatergic and GABAergic neurons [128].

On the other hand, synaptic transmission modulated by nicotinic receptors (nAChRs) is also sensitive to inhibition by Pb [129]. Pb blocks the action potential-dependent transmitter release mediated by activation of α4β2 and α7 nAChRs via protein kinase C in hippocampal neurons [130], and there is also evidence of the direct interaction of Pb with these receptors [129,131]. According to these data, recently, it has been reported in mice that a chronic Pb exposure during the lactation period raises brain levels of kynurenic acid (KYNA). KYNA is an antagonist of NMDAr and a negative allosteric modulator of α7 nAChRs. This study provided evidence of the involvement of tryptophan catabolism and kynurenine pathway as a new mechanism of Pb toxicity affecting neurotransmission and impairing cognitive performance [132].

Therefore, scientific research has provided the diversity and complex mechanisms by which Pb impair cognitive functions (Figure 2). The challenge for the new research is to consider these mechanisms as dependent factors, and that they could converge and influence neuronal activity simultaneously and, as a consequence, be critical in the shaping of functional neural circuits during development and throughout adult life.

### 3.2. Behavioral and Structural Alterations Induced by Pb Exposure in Animal Models

In animal models, neurobehavioral deficits induced by prenatal, preweaning, and postweaning Pb exposure persist until adulthood. It has been described that Pb exposure alters the locomotor activity [133,134], increases aggressive behavior [135], and causes motor coordination impairment [136], response inhibition deficits, deficient task solving capacities [137,138], and learning and memory impairment [132,139,140,141].

A study carried out in Cynomolgus monkeys exposed to Pb from birth to 200 days of age showed impairments on a discrimination reversal task (nonspatial form discrimination, nonspatial colour discrimination, and nonspatial form discrimination) at three years of age with blood Pb concentration of 11–13 µg/dL at the time of testing [142]. In a different experimental approach, *Macaca fascicularis* monkeys were exposed to Pb acetate (2000 µg/kg/day) during early life (100–270 days); then, they were tested during infancy (2–3 years old) on a series of nonspatial discrimination reversal tasks that resulted in the lack of or only marginal deficits. However, as adults, they presented performance alterations on a differential reinforcement of low rate (DRL) schedule of reinforcement task, and four or five treated monkeys were also unable to learn the visual discrimination task without a remedial training procedure [143,144]. These results confirm that continuous exposition to Pb during early life impacts cognitive performance in adulthood. Additionally, Muller et al. evaluated how a single dose of Pb administered into the yolk sac on the fifth incubation day of the *Gallus domesticus* can affect the behavior and the brain tissue in the first post-natal week. Lead exposure (28 µg Pb acetate/100 µL in the yolk sac) induced Pb deposition in both mesencephalon and cerebellum of newborn chicks. Lead-induced deficits on motor behavior showing abnormal movements in chicks during the first postnatal week were found to be related to Pb deposition in the cerebellar tissue during embryonic development [145]. In line with these findings, Petit and Alfano (1979) demonstrated that the hyperactivity and deficits shown in a maze performance test in rats exposed to PbCO_3_ (high Pb 4%) during PD1 to PD25 was absent in rats exposed to low Pb (0.4%), showing no differences with control in both tasks. However, both groups (low and high Pb) showed a deficit in passive avoidance cognition performance test; interestingly, when these rats were exposed to environmental enrichment, low Pb-exposed rats increased passive avoidance latencies above control values, but it had no effect in high Pb-exposed rats [146].

As mentioned above, cognitive alterations induced by Pb are related to its accumulation, mainly in the astroglia [147]. The morphological changes in the fine structure of neurons and their synaptic connections have been observed in brains exposed to Pb during the prenatal and early life, which is also associated to a reduction in brain weight, reduction in cerebellum size, and a reduction of thickness of both cerebral cortex and hippocampus [148,149]. Postnatal Pb exposure affects cortical neural ontogenesis [150] and produces deficient dendritic development in both cerebellar Purkinje [151] and neocortical pyramidal cells [152] resulting in a reduction in the number and rate of maturation of neocortical synapses [146]. Pb also affects myelination; rats exposed to Pb pre- and neonatally showed abnormal myelination in the cerebral cortex and vascularization in cerebral cortex and cerebellum [153]. Lead damage has been proven to be even at the ultrastructural level since hippocampal neurons Pb-exposed showed elongated and shrunken mitochondria, irregular nuclei, and swollen synapses with thickened vesicles in the presynaptic cleft. These changes were associated with alterations in synaptic proteins including syntaxin-1, synaptotagmin-1, and SNAP25 [154,155]. Long-term effects in structural plasticity within the hippocampus have also been described in rats exposed to Pb-acetate (0.2%), from the gestation until PD87, where a decrease in the survival of newly generated granule cells in dentate gyrus (around 50% vs. control) was found to be related to a reduction capacity for structural plasticity in the adult hippocampus. However, when rats were exposed to Pb during gestation to PD21, no significant changes were found (around 20% vs. control), suggesting that chronic Pb exposure is necessary to affect neurogenesis [156]. Additionally, Jaako-Movits and coworkers showed that rats PD80 exposed to Pb acetate (0.2%) in drinking water, from PD1 to PD30, increased the level of anxiety and inhibition of contextual fear conditioning. Moreover, when neuronal proliferation was tracked administering BrdU, the number of BdrU-positive cells in the dentate gyrus decreased in the Pb group (around 23%) compared to the control group. Approximately 28.7% of the BdrU positive cells were colocalized with calbindin, a calcium binding-protein and a marker for mature neurons, in the Pb group, whereas the control expressed 40.6%. Additionally, a significantly higher proportion of BdrU positive cells of young not fully differentiated neurons and a higher proportion of newborn cells differentiated into astroglia were found in the Pb group (35.4%) compared to the control group (12%). These findings show that early Pb exposure affects neurogenesis, alters the pattern of differentiation of newly born hippocampal cells, and suggests that these alterations may contribute to behavioral and cognitive impairments characterized in adulthood [157].

### 3.3. Redox and Energetic Imbalance Induced by Pb Toxicity

One of the mechanisms attributed to Pb neurotoxicity involves the generation of reactive oxygen species (ROS) and the reduction of endogenous antioxidants. This heavy metal can inactivate various antioxidant enzymes through its binding to thiol functional groups, contributing to exacerbate a pro-oxidant environment. A recent meta-analysis study that included 108 articles showed that mice are more susceptible than rats to the oxidative stress induced by Pb exposure. Additionally, this study demonstrated that an increasing Pb concentration shows a positive correlation with glutathione disulfide (GSSG), ROS, and glutathione peroxidase (GPx) and a negative correlation with glutathione reductase (GR), superoxide dismutase (SOD), and glutathione S-transferase (GST) (enzymes involved in the control of redox status). Thus, it confirmed that a high dose of Pb resulted in an oxidant environment and that the damage generated by this heavy metal is time and dose-dependent [158].

In this context, it was observed that prenatal exposure to Pb acetate, beginning on Day 5 of gestation until 14 days after birth, causes Pb accumulation in the brain and cerebellum of newborn rats. Additionally, rats exposed to Pb during gestation and in early postnatal days showed an increased brain lipid peroxidation levels in male and female newborns (2 and 2.5 folds, respectively) [159]. Moreover, the brain antioxidant enzyme activities of SOD1, SOD2, GPx, and CAT were exhibited after exposure to 0.3% Pb acetate in drinking water during gestation and until 21 postnatal days (PD) [160]. An additional study found a decrease in SOD activity and an increase in GPx activity in the pups forebrain cortex, cerebellum, and hippocampus in a dose-dependent manner after dams were exposed to Pb (50, 100, and 500 ppm) [161]. Pb also decreases antioxidants enzyme activities due to the high Pb affinity to sulfhydryl groups or metal cofactors critical for these enzymes; for example, Pb competes and replaces Cu and Zn in their binding sites [162,163]. Experimental studies using juvenile rockfish *Sebastes schlegelii* exposed to Pb dietary (0, 120, and 240 mg/L) for four weeks increased SOD and GST activity, while GSH levels decreased in the liver and gills [164]. In Drosophila, it was demonstrated that Pb exposure induces iron deficiency, and this effect was modulated by ROS production, since iron reduction was mitigated when ROS were diminished by N-acetylcysteine addition [165]. 

On the other hand, it has been shown that younger astroglia accumulates more Pb than old astroglia and neuronal cells; however, neurons are more susceptible to Pb toxicity than astrocytes [166,167]. Recently, the response of primary microglia and astrocytes to Pb toxicity was evaluated. In this study, both microglia and astrocytes were incubated for 24 h with Pb (15–100 µM) and showed decrease in cell viability; however, Pb intracellular levels were higher in astrocytes than in microglia. Intriguingly, microglia but no astrocytes showed a significant increase in ROS production and GSH levels reduction [168]. Additionally, as part of an antioxidant response in microglia, the nuclear factor erythroid 2-related factor 2 (Nrf2) protein (a regulator of cytoprotective factors against oxidative stress) increased in a dose-dependent manner, along with mRNA expression and protein levels of heme-oxygenase-1 (HO-1) and quinone oxidoreductase-1 (NQO1) upregulation after Pb exposure. Moreover, when Nrf2 was silenced in microglia and then exposed to Pb, ROS production was higher and the cell viability decreases, suggesting that Nrf2 plays a protective role against Pb toxicity in microglia [168]. Parallelly, oxidative stress induced by Pb exposure has been shown, and, regardless of the activation of the Nrf2-ARE signaling pathway, oxidative DNA damage occurs and is exacerbated by promoter methylation of DNA repair genes in human lymphoblastoid TK6 cells [169]. Nrf2 protective role against oxidative stress induced by Pb has also been demonstrated in SH-Sy5Y cells. It was observed that Nrf2 content increased in the nucleus and decreased in the cytoplasm after 1 h of Pb exposure in SH-Sy5Y cells leading to increased Nrf2-ARE binding activity. Additionally, overexpression of Nrf2 in these cells exposed to Pb diminished ROS production and apoptosis while cell viability was increased; meanwhile, the silencing Nrf2 decreased Nrf2-regulated genes and protein expression in response to Pb [170].

Mitochondrial dysfunction is also involved in Pb toxicity. It has been found that liver mitochondria exposed both to different times and concentrations of Pb (0–80 µM) increases ROS production and mitochondria lipid peroxidation while GSH levels decrease in a concentration and time-dependent manner. Furthermore, it has been shown that Pb intoxication induces alterations in the mitochondrial energy metabolism decreasing ATP levels and oxygen uptake as well as decreasing cytochrome oxidase, succinate dehydrogenase and NADH dehydrogenase activities [171]. Besides, it was observed that Pb decreases the enzyme activities of the mitochondrial respiratory chain complexes, being the complex III the most susceptible to Pb exposure through a decrease in ATP levels, mitochondrial permeability transition pore (MPTP) opening and the release of cytochrome c [172]. Moreover, Pb causes ATP reduction affecting the physiological functions of Na^+^-K^+^-ATPase activity and Ca^+2^-ATPase activity [173]. In this context, it has been shown in primary cultured cerebellar granule neurons obtained from rats exposed prenatally to Pb that Pb decreases ATP levels and mitochondrial membrane potential, inhibits Na^+^/K^+^ ATPase activity, and increases intracellular and mitochondrial ROS synthesis [174]. It was recently shown that peroxisome proliferator-activated receptor-γ coactivator 1α (PGC1α) regulates the mitochondrial biogenesis and dynamics in neurons exposed to Pb. PGC1α expression increases after Pb exposure, and it was associated to structural alteration in mitochondrial filaments and dysregulation of genes responsible for mitochondrial dynamics. Dysregulation of PGC1α expression renders more susceptible cells to Pb, leading to mitochondrial fragmentation and cell death [175]. These mitochondrial alterations could also be related to the Pb ability to influence calcium pathways within the endoplasmic reticulum through BAP31 protein. BAP31 is an integral membrane protein located in the endoplasmic reticulum where it is cleaved by caspase-8, and then it stimulates Ca^2+^-dependent mitochondrial fission and enhances pro-apoptotic signals through the interaction between endoplasmic reticulum and mitochondria. BAP31 protein levels increase after Pb exposure, and BAP31 knockdown reduces the cell signaling death pathways induced by this metal [175,176]. Moreover, a recent study showed that Pb induces apoptosis via the mitochondria pathway in both chicken brain tissues and embryonic neurocytes. This heavy metal can decrease mRNA and protein expression of Bcl-2 (an anti-apoptotic protein) while increase mRNA and protein expression of p53, Bax, Cyt C, and caspase-3 [177]. 

Furthermore, Gu and co-workers showed that autophagy disruption is another target in Pb neurotoxicity [178]. It has been shown in PC12 cells that Pb increases the expression of LC3-II, an important marker for autophagy, and shows a positive correlation with a decrease in cell viability. Additionally, SQSTM1/p62 (an autophagic substrate) increases after Pb exposure, suggesting autophagy inhibition. Autophagy inhibition by Pb is evidenced by autophagosomes accumulation, since it interferes with the autophagic flux (autophagosomes formation, fusion of autophagosomes with lysosomes, and the breakdown and release of resulting molecules back into the cytosol) and directly with the number, acidification, or size of the lysosomes, leading to the inhibition of the genesis and the critical function of lysosomes in the autophagic process [178].

As mentioned above, Pb can substitute calcium and act as a ligand for the calcium-binding sites and disrupt the downstream signaling effector proteins, mainly protein kinase C and calmodulin, contributing to its neurotoxicity [179]. Indeed, experimental studies in broken cell preparation of immature brain micro-vessels have shown that Pb activates protein kinase C (modulator of cellular proliferation and differentiation) [180] and inhibits Na^+^/K^+^-ATPase, thus interfering with energy metabolism. Additionally, it was shown that Pb rise the intracellular free calcium in a dose-dependent manner and is mediated through the activation of PKC. Interestingly, in this study, Pb was proven to be a potent activator of PKC at concentrations 3000 times lower than those of Ca^2+^; these observations suggest that toxic sequelae of Pb exposure may include alterations in the PKC signal transduction system within the cells [181]. Recently, it has been demonstrated that Pb binds downstream regulatory element antagonist modulator (DREAM) protein displacing Ca^2+^ from the EF-hands because of its higher affinity (50 times more than Ca^2+^) for the Ca^2+^-binding sites in DREAM. Pb modulates DREAM interaction with intracellular partners, controlling gene expression for apoptosis, learning, and memory process [182]. Likewise, Pb binds to human phospholipid scramblase 3 (hPLSCR3), another calcium-dependent mitochondrial membrane protein involved in the apoptosis signaling. The Pb-binding to hPLSCR3 in the calcium-binding motif presents higher affinity than Ca^2+^ and is responsible for phospholipid translocation to the outer mitochondrial membrane [183]. 

On the other hand, it has been shown that Pb exposure (pre- and postnatal) increases the expression and activity of glycogen synthase kinase 3β in the rat brain and decreases the expression of glycogen synthase and glycogen phosphorylase, contributing to the dysregulation of glycogen metabolism, thereby reducing glucose availability and triggering brain energy metabolism alterations [184,185]. 

Several in vitro and in vivo experiments shown that exposure to Pb alters both the calcium and redox homeostasis, impair mitochondrial function, and consequently alter brain ATP synthesis. These factors, related intrinsically, can converge and could, in turn, limit many cellular functions including neurotransmitter vesicular release, thus impacting the storing and release of neurotransmitters and then compromising cognitive and behavioral processes (Figure 2).

### 3.4. Strategies Used against Pb Neurotoxicity in Experimental Models

Pb toxicity has many targets and different experimental strategies have been used to decrease its toxicity. Many of these strategies are focused on modulating the redox environment, and some of them have an impact on behavioral and cognitive performance (Table 3). In this context, El-Missiry reported that rats receiving an intramuscular injection of Pb acetate (10 mg/kg) for seven days but pre-treated with melatonin (30 mg/kg) reduced the inhibitory effect of Pb on glutathione reductase, glutathione-S-transferase, SOD, and CAT, as well as in nonenzymatic antioxidants such as total sulfhydryl groups and glutathione (GSH) [186]. 

An additional study showed that the simultaneous administration (21 days) of melatonin to rats exposed to Pb attenuated the increase in lipid peroxidation products and restored GSH levels and SOD activity, reduced the morphological damage within the hippocampus and the striatum, and prevented the neuronal density reduction induced by Pb exposure [206]. The beneficial effects of calcium and zinc were used as strategy against the oxidative stress induced by Pb exposure. Mice were exposed to 0.2% Pb during lactation and supplemented with calcium or zinc in the same period; those treated with calcium or zinc showed recovery in the antioxidant enzymes activity and decreased both lipid peroxidation and free radicals formation [163]. Additional antioxidant strategies using N-acetylcysteine (NAC) administration following Pb exposure resulted in a significant decrease in the brain lipid peroxidation (~70% compared to the Pb-treated group). This effect in lipid peroxidation was attributed to NAC’s ability to enhance the antioxidants defenses by acting as a major precursor for thiols groups as it gets deacetylated to cysteine. Additionally, NAC increases the CAT and SOD activity [162]. In concordance, the *Centella asiatica* leaf extract shows protection in a model of behavioral impairment induced by Pb exposure during lactation in rats [189].

Many molecules have been demonstrated to prevent some of the toxic mechanisms induced by Pb; however, a combination of different treatments should be considered, as this heavy metal has many targets and the intensity of its harmful effects depends on the time duration and doses, administration route, and the experimental animal model used. In future studies, it would be pertinent to evaluate molecules that affect more than one target for Pb neurotoxicity and to reinforce these studies with behavioral tests that allow determining if the effect of the therapy mitigates the cognitive impairment induced by this heavy metal.

## 4. Conclusions

As we shown throughout this review, cognitive alterations induced by Pb are evident consequences of its toxicity; however, Pb toxicity also involves diverse converging mechanisms that are closely related, which lead to cellular dysfunction and neuronal death that ultimately trigger cognitive alterations. This suggests that new research should consider that these mechanisms are acting synergistically within the same biologic pathways to induce neurotoxicity and the challenge is to find therapeutic strategies that have an impact on more than one of these mechanisms to prevent and reduce the cognitive dysfunction induced by Pb exposure.

## Figures and Tables

**Table 1:** Epidemiological studies of association between lead levels and cognitive impairment by different ages of exposure.

Population (Age Range)	Study Design and Subjects	Location	Lead Levels	Outcomes	Reference
*Children*					
2–4 years	Cross-sectional study; 76 children. Gesell Developmental Scale: *t*-test comparisons. The Achenbach Child Behavior Checklist (CBCL): χ^2^ test. Scores on each behavior factor and the total behavioral score were analyzed by the rank-sum test.	Xi’an, China	Blood: 4–246 µg/L	↓ Adaptative behavior, gross motor performance, fine motor movements, language development, and individual social behavior. ↑ Depression, abnormal behavior, aggressions, social withdrawal, sleep problems, and destruction.	[11]
2–5 years	Cross-sectional study; 201 children. Child Behavior Checklist (CBCL): χ^2^ test and *t*-test comparisons. Total Behavior Problem Score (TBPS) based upon the percentile ranking of raw scores and in the clinical range (90th percentile). Multiple regression analysis for the likelihood ratio and influential factor on the TBPS was measured from the mothers using the Center for Epidemiologic Studies Depression Scale (CES-D).	Baltimore, United States	Blood: 2–30 µg/dL	Troublesome behaviors (aggression, sleep problems, and somatic problems) Increase in externalizing behaviors (motor activity, nonadaptive behaviors, conduct problems, inattentiveness, passivity, and hyperactivity)	[12]
8–9 years	Cross-sectional study; 167 children. Wechsler Intelligence Scale for Children (WISC), Göttinger Formreproduktions-Test (GFT), Bender Gestalt-Test (German version), Benton Test, Diagnostics for Cerebral Damage Test and Wiener Reaction Device. Duisburg study t tests for correlating samples. Stolberg sample associations were tested using of stepwise multiple regression analysis.	Duisburg city and Stolberg city, Germany	Baby teeth: 1.4–38.5 µg/g Blood: 6.8–34 µg/100 mL	Deterioration of visual-motor integration IQ deficits (5–7 points) Disturbance of reaction performance Deficits in verbal IQ (4–7 points)	[13]
6–12 years	100 children Wechsler Intelligence Scale for Children, 3rd edition (WISC-III) Spearman’s rank correlation, logistic and linear regressions to test independent predictors for impairment of cognitive function and the relationship between blood lead levels and cognitive function. Receiver operating characteristic (ROC) curve was used to calculate the best cut-off value of blood lead levels (based on the highest sensitivity with the lowest false-positive results) above which the majority of the children have cognitive dysfunction.	Cairo, Egypt	Blood: 3–28 µg/dL	↓ IQ in 15 points (mainly in verbal subsets, arithmetic and digit span) Poor scholastic achievement Cognitive dysfunction (flat affect, slow, and delayed responses)	[14]
*Adults*					
50–60 years	Cross-sectional study; 53 adults. Wechsler adult intelligence scale-revised (WAIS-R), Wechsler memory scale (WMS), a test of attention and visuomotor tracking (trail making), test of verbal fluency (FAS), test of non-verbal reasoning (Raven progressive matrices), test of motor speed (finger tapping) and inventory of current mood (POMS). Wilcoxon signed ranks test, used to compare matched pairs of subjects exposed to lead and controls; and the Mann–Whitney test, used to compare entire groups. χ^2^ test was used to evaluate the distribution of categorical frequencies.	Boston, United States	Blood: 60–120 µg/dL	Difficulties in attention and executive functioning, reasoning, and short-term memory. Attenuation of the ability to learn new information. Problems with attention and concentration Reduced ability to do more than one activity at the same time. Impaired ability to organize information or steps in a procedure. Increased difficulty in arriving at solutions for problems.	[8]
50–70 years	Cross-sectional study; 1033 adults. A battery of 20 cognitive test results was standardized and collapsed into 7 cognitive domain scores. All 7 domain scores were standardized for direction so that a negative regression coefficient indicated worse performance. χ^2^ test for interaction between tibia lead, Neighborhood Psychosocial Hazards Scale (NPH). NPH scale and the 7 domains of Cognitive function in the Baltimore Memory study. Multilevel regression models were used to account for the nesting of persons within neighborhoods.	Baltimore, USA	Tibia 18.8 ± 11.6 µg/g	Affectations in language and executive function	[1]
*Male workers*					
22 years	Case report	India	Blood: 128.3 µg/dL	Aggressiveness	[15]
39–50 years	Cross-sectional study 47 adults exposed to Pb for 11.7 ± 9 years. Modified version of the Wisconsin card sorting test, the block design test, the visual recognition test, choice reaction, simple reaction, and digit symbol substitution. One-tailed *t*-test for independent samples was used for the following tasks: block design and visual recognition tests, simple reaction time, and digit symbol substitution. The results of the choice reaction were analyzed by multivariate analysis of variance. Since scores of the Wisconsin test were not distributed normally, they were analyzed by one-tailed Mann– Whitney test. Because of multiple univariate testing, Bonferroni correction was applied.	Germany	Blood: 30.8 + 11.2 µg/100 mL	Neurobehavioral performance poorer than control in categories such as executive functions (visual recognition), short-time memory, and visuospatial abilities.	[16]
25–67 years	Cross-sectional study: 100 adults exposed to Pb for 1–7 years. WHO neurobehavioral core test battery. Multiple linear regression of neurobehavioral function in workers and lead exposure indices. Correlation coefficients (Pearson r) between blood lead concentration and covariates Analysis of covariance for dichotomous exposure variables.	Venezuela	Blood: 9–60 µg/dL	↓ results on Wechsler adult intelligence subtests. ↓ Simple reaction time. Mood indicative of depression. Anger hostility, fatigue, and confusion.	[17]
71 years	Case report Twins	Boston, United States	Blood: 15–125 µg/dL Patella: 119–343 µg/g Tibia: 79–189 µg/g	Verbal/language abilities and working memory/executive function lower than average (worse in twin 1) Deficits in short-term memory function (in learning and retention of new information) Bad manual motor control	[18]
*Female workers*					
55–65 years	Cross-sectional study: 31 adults exposed to Pb for 1.4–20.7 years. N-back working memory paradigm. Mean values of continuous variables were compared using the Student’s *t*-test. Pearson correlation analyses between mean percentage changes of activated brain regions and working memory performance. The effects of blood lead on percent signal change by multiple regression analysis.	Korea	Blood: 0.88–13.5 µg/dL	Decreased working memory (in-back memory task). Decreased activation in the dorsolateral prefrontal cortex, ventrolateral prefrontal cortex, pre-supplementary motor areas, and inferior parietal cortex	[19]

**Table 2:** Biomonitoring of human exposure to Pb in pregnant women and lifespan development.

Population	Study Design and Subjects	Study Period and Location	Sample	Lead Levels	Reference
*Pregnant woman*					
	Cross-sectional study 53 female patients 29.11 ± 4.77 years	2007–2008 Szczecin, Poland	Blood Milk	1.290 ± 0.578 μg/dL 0.174 ± 1.15 μg/dL	[41]
	Cross-sectional study 91 female patients	Five months Ontario, Canada	Blood Milk	22.9 ± 12.5 μg/L 2.08 ± 1.67 μg/L	[42]
	Cross-sectional study 156 female patients 29–34 years	November 2015–December 2016 Beijing, China	Blood Cord blood	23.1 ± 21.2 μg/L 14.2 ± 7.6 μg/L	[43]
	Prospective case-control study 21 pregnant woman 26.5 ± 5.5 years	October 2016 to April 2017 Ankara, Turkey	Blood	12.3 μg/L	[44]
	Cross-sectional study 41 patients (29 ± 6 years)	2003–2004 Oroya City, Peru	Blood Cord blood	27.4 ± 15.6 μg/L 19.0 ± 12.6 μg/L 319 ± 215 µg/100 g	[45]
*Newborns*					
	Cross-sectional study 53 newborns	2007–2008 Szczecin, Poland	Blood	~1 ug/dL	[41]
	Cross-sectional study 91 infants (0–4 months)	Five months Ontario, Canada	Cord Blood	20.8 ± 16.7 μg/L 16.7 ± 10.4 μg/L	[42,46]
	Birth cohort study 79 newborns	September, October 2008 Terai, Nepal	Cord blood	31.7 + 35.36 μg/L	[47]
*Children*					
	Cross-sectional study 1–5 years	2011–2016 South Carolina, United States	Blood	0.27–20.4 μg/dL	[48]
	Cross-sectional study 120 children Age 1–36 months	2010–2011 Shandong, China	Blood	42.18 ± 12.13 μg/L	[49]
	Cross-sectional study 2397 children 1–4 years	2013 Sao Paolo, Brazil	Blood	6.3–8.1 μg/dL	[50]
	Cross-sectional study 130 children 0.33-5.8 years	2003–2013 Ecuador	Blood	29.4 ± 24.3 µg/dL	[51]
	Cross-sectional study 561 children 3 months–9 years	July–August 2017 Kabwe, Zambia	Blood	5–100 μg/dL	[52]
	Cross-sectional study 301 children 6 months–16 years	2003–2016 Refugees in the United States	Blood	1–27 μg/dL	[53]
*Adults*					
	Case control study 454 adults (35–65 years)	2009 Florence	Blood	86.1 μg/L	[54]
	Cross-sectional study 15,123 residents (≥16 years).	2009–2015 Missouri	Blood	1.5–≥25 μg/dL	[55]
	Cross-sectional study 30 participants 70.4 ± 9.1 years	2009–2010 Shanghai, China	Blood Bone	1.0–23.2 μg/dL 0.9–15 μg/g	[56]
	Cross-sectional, case control 80 adults (male and female; 15–47 years)	January 2017–July 2018 India	Blood	38.02 ± 19.92 μg/dL	[57]
	Cross-sectional study 171 male adults 40.9 ± 8.25 years	2007–2008 Poland	Blood	42.9 ± 6.3 μg/dL	[58]
	Cross-sectional study 52 male adults (39 ± 9 years)	2019 Tunisia	Blood Urine Hair	101–535.3 μg/dL 15.8–72.0 μg/dL 2.2 ± 0.1 μg/g	[59]
	Cross sectional study 139 adults (male and female; 16–67 years)	2018 Iraq	Blood	5.77 μg/dL	[60]

**Table 3:** Therapies used against Pb neurotoxicity in experimental models.

Animal Model.	Doses	Behavioral Alterations/Cognitive Impairment	Biochemical and Morphological Alterations	Therapy	Reference
Male Wistar rats	20 mg PbAc/kg i.p. for 14 days	↓ Rotarod activity ↓ Time in open field test ↑ Time in adhesive removal test ↑ Escape latency time in Morris water maze ↑ Immobility time in the forced swim test ↓ Grip strength time in string test	↑ Lipid peroxidation, nitric oxide, and protein carbonile ↓ Superoxide dismutase, catalase, glutathione peroxidase activity, glutathione reductase, and glutathione-S-transferase activity ↓ Glutathione, vitamin C and E ↓ Na^+^/K^+^ ATPase activity ↓ Acetyl cholinesterase ↓ Survival cell number ↑ Bax/Bcl-2 ratio ↓ Mitochondrial cytochrome c ↑ Cytosolic cytochrome c	Morin (40 mg/kg) orally 2 h after the administration of PbAc for 14 days). Attenuates all the behavioral and biochemical alterations induced by PbAc	[187]
Pregnant Wistar rats	0.2% PbAc, daily from the 5th day of gestation until weaning (PD21).	↓ Rotarod activity ↓ Number of rearing	↑ TBARS ↓ SOD activity ↓ GPx activity ↓ Cerebellar Purkinje cell number	Melatonin (10 mg/kg) once daily through oral gavage during the gestational and lactational period. Attenuates the effect on behavioral and biochemical alterations induced by Pb.	[188]
Wistar rats	0.015% PbAc in drinking water from gestation until PD21	↓ Exploratory, locomotory, cognitive impairment ↑ Analgesic reaction time	↑ Lipid peroxidation ↓ GSH levels ↓ The activity of SOD, catalase, GPx and glutathione reductase	Extract of *Centella asiatica* (200 mg of crude/kg body weight/day) from PD21 to PD60. Prevents the behavioral and brain redox alterations induced by Pb.	[189]
Wistar rats	0.2% PbAc in drinking water from gestational day 6 to PD21		↓ Activity of serum ceruloplasmin oxidase (Cp), Mn-SOD, Cu/Zn-SOD, GPx, CAT, and xanthine oxidase ↑ Malondialdehyde (MDA) levels increased in the cerebellum and hippocampus	Calcium supplement (0.02% in Pb–water) Reversed Pb toxicity	[190]
Sprague Dawley rats	0.2% PbAc in drinking water during the gestational period		↑ Apoptotic cell deaths ↓ The number of Purkinje cells in the cerebellum ↓ Synaptophysin and NMDAr subtype 1 density	Ascorbic acid (100 mg/kg) Reduces Bax and apoptotic neuronal death and prevents the impairment in cerebellar synaptic proteins.	[191,192]
Wistar rats	20 mg PbAc/kg i.p. for 7 days		↑ Cortical lipid peroxidation, nitrate/nitrite levels, and inducible nitric oxide synthase expression ↓ Glutathione content, superoxide dismutase, catalase, glutathione peroxidase, glutathione reductase activity and mRNA expression ↓ Nuclear factor erythroid 2–related factor 2 (Nrf2) and hemoxygenase-1 (HO-1) expression. ↑ The cortical levels of serotonin, dopamine, norepinephrine, GABA, and glutamate, ↓ The level of ATP	Coenzyme Q10 (10 mg/kg i.p./7 days) Restores the balance between oxidants and antioxidants, inhibiting the apoptotic cascade, and modulating cortical neurotransmission and energy metabolism.	[193]
Male Wistar rats	0.2% PbAc daily for 3 months		↑ Malondialdehyde and total oxidant status in plasma ↓ The excitatory postsynaptic potentials slope and the population spike amplitude	Vitamin C (150 mg/kg, daily for 3 months) Increases total antioxidant capacity inhibiting the effects of Pb	[194]
Sprague-Dawley (SD) rats	0.2% PbAc during the gestational period to PD43	Induces working memory deficits	↓ Dendritic spine density ↓ SOD and GPx activity and expression in the hippocampus.	Kiwi fruit (12 mg/kg daily from 7 to 9 weeks old) Alleviates cognitive deficits and restores the antioxidant environment	[195]
Male Sprague–Dawley rats	200 ppm PbAc for 8 weeks	Impaired spatial reference memory	↑ Apoptotic cell death and the expression of Bax ↓ Bcl-2 protein expression	Genistein (1 mg/kg/day) Diminishes impairment in cognitive function and protects neurons from Pb toxicity. N-acetyl-l-cysteine (NAC; 1 mg/kg/day) Prevents cognitive impairment and reduced cell death.	[196]
Wistar rats	0.2% PbAc from gestation to PD21	Induces learning and memory deficits	↓ GSH content ↓ SOD activity ↑ MDA	Allicin (30 mg/kg allicin twice a day for 21 days) Alleviates learning and memory deficits and reverts biochemical parameters altered by Pb.	[197]
*Caenorhabditis elegans*	100 µM of PbAc for 24 h	Decline of locomotion behaviors (frequencies of body bends, head thrashes, and reversal)	↑ Intracellular ROS production	Se(IV) (0.01 µM) Ameliorates locomotion behavioral alterations induced by Pb. Decreases intracellular ROS and protects sensory neurons from Pb.	[198]
Sprague Dawley rats	100 and 400 ppm PbAc for 15 days		↑ Synaptosomal lipid peroxidation, protein carbonylation and 3-nitrotyrosine levels ↓ Thiol content Inhibits complexes II, III and IV of the mitochondrial respiratory and decreases ATP and transmembrane potential	MitoQ (500 μM for 15 days). Prevents the oxidative damage induced by Pb. Alleviates mitochondrial dysfunction	[199]
Wistar rats	0.2% PbAc in drinking water		Impairment of synaptic plasticity	Quercetin (30 mg/kg, for 7 days) Prevent Pb alterations.	[200]
Male Sprague Dawley rats	75 mg/kg PbAc for 4 weeks	Anxiety and Aggression	↓ Serotonin ↓ Reduced glutathione levels, antioxidant enzyme activity ↑ Lipid peroxidation and brain protein contents	Ascorbic acid (40 mg/kg) Attenuates oxidative stress and abnormalities in behavior induced by Pb. Tryptophan (20 mg/kg) Ameliorates altered neurobehavior induced by Pb, with no significant effect on Pb induced oxidative stress in the brain.	[201]
ICR mice	1% PbAc in drinking water for 38 days	Depression Memory loss	↓ AChE activity ↑ Malondialdehyde levels	Xanthone derivative of *Garcinia mangostana* (100 and 200 mg/kg) Increases AChE activity and decreases lipid peroxidation Ameliorates depression-like effect and memory loss induced by Pb	[202]
Wistar rats	0.2% PbAc solution was injected intraperitoneally		Impaired synaptic transmission and plasticity in the hippocampus ↑ SOD activity and malondialdehyde ↑ Intracellular calcium	Monosialoganglioside (100 μg/mL microinjection into hippocampus). Prevents the impairments of synaptic plasticity, antioxidant system function, and intracellular calcium levels	[203]
Wistar rats	0.2% PbAc for 4 weeks	Affects spatial reference memory ability. Increases in the scape latency	↓ Body weight ↓ Decrease of antioxidant activities and BDNF content	Tanshinone IIA (4 and 8 mg/kg) Improves antioxidant activities by increasing SOD activity and GSH and decreasing MDA levels Prevents the impairment in the spatial reference memory.	[204]
ICR mice	drinking water (1 g/L), 38 days	Impairment memory	↓ AchE activity	*Thunbergia laurifolia* (100 and 200 mg/kg/day). Attenuates cognitive impairment and increased AchE activity.	[205]
